# Rapid and Targeted Introgression of Genes into Popular Wheat Cultivars Using Marker-Assisted Background Selection

**DOI:** 10.1371/journal.pone.0005752

**Published:** 2009-06-01

**Authors:** Harpinder S. Randhawa, Jasdeep S. Mutti, Kim Kidwell, Craig F. Morris, Xianming Chen, Kulvinder S. Gill

**Affiliations:** 1 Department of Crop and Soil Sciences, Washington State University, Pullman, Washington, United States of America; 2 USDA-ARS Western Wheat Quality Laboratory, Washington State University, Pullman, Washington, United States of America; 3 USDA-ARS, Wheat Genetics, Quality, Physiology, and Disease Research Unit and Department of Plant Pathology, Washington State University, Pullman, Washington, United States of America; Purdue University, United States of America

## Abstract

A marker-assisted background selection (MABS)-based gene introgression approach in wheat (*Triticum aestivum* L.) was optimized, where 97% or more of a recurrent parent genome (RPG) can be recovered in just two backcross (BC) generations. A four-step MABS method was developed based on ‘Plabsim’ computer simulations and wheat genome structure information. During empirical optimization of this method, double recombinants around the target gene were selected in a step-wise fashion during the two BC cycles followed by selection for recurrent parent genotype on non-carrier chromosomes. The average spacing between carrier chromosome markers was <4 cM. For non-carrier chromosome markers that flanked each of the 48 wheat gene-rich regions, this distance was ∼12 cM. Employed to introgress seedling stripe rust (*Puccinia striiformis* f. sp. *tritici*) resistance gene *Yr15* into the spring wheat cultivar ‘Zak’, marker analysis of 2,187 backcross-derived progeny resulted in the recovery of a BC_2_F_2∶3_ plant with 97% of the recurrent parent genome. In contrast, only 82% of the recurrent parent genome was recovered in phenotypically selected BC_4_F_7_ plants developed without MABS. Field evaluation results from 17 locations indicated that the MABS-derived line was either equal or superior to the recurrent parent for the tested agronomic characteristics. Based on these results, MABS is recommended as a strategy for rapidly introgressing a targeted gene into a wheat genotype in just two backcross generations while recovering 97% or more of the recurrent parent genotype.

## Introduction

Many factors can shorten the commercial life-span of a cultivar including sudden population shifts and/or mutations in ever-evolving, dynamic pest populations that circumvent deployed resistance genes, or changes in consumer preference, that leave a cultivar unmarketable. Rapid introgression of single genes in a targeted and identity-preserved manner is essential to alleviate these constraints on the continued success of a cultivar, to protect and increase yield potential, and to benefit from newly available value-added genes in a timely manner.

Single gene introgressions are routinely performed by repeated backcrosses (BC) in an attempt to transfer the targeted gene into the recurrent parent genome (RPG). Without making a distinction between carrier and non-carrier chromosomes, simulations assumed a 50% reduction in donor genome with each backcross cycle, and thus predicted that 99.2% of the RPG would be recovered after the sixth backcross generation [1]. Backcrossing only a few plants selected at random during each cycle is not expected to yield the simulated RPG recovery rate for the carrier chromosome due to the low probability of selecting double recombinants around the target gene. Around the *Tm-2* gene of tomato, for example, linkage drag (donor chromatin linked to the target gene) was 51 cM even after 11 BC generations, which is equivalent to nearly half of the donor chromosome [2]. For the non-carrier chromosomes, the probability of recovering a plant with all recurrent parent type chromosomes is equally low. Therefore, it is highly unlikely to attain the predicted RPG recovery without genotyping a large BC population in order to identify a plant carrying the maximum proportion of RPG. Marker-assisted selection (MAS) is ideal for selecting both a target gene (foreground selection), as well as recurrent parent genotype for the rest of the genome (marker-assisted background selection, MABS) [3].

Computer simulations in tomato predicted that an MABS approach can recover up to 99% of the RPG in just three BC cycles compared to the 100 cycles required without marker selection [3]. By screening 255 plants with 61 markers during the transfer of CryIA(b) *Bt* gene to maize inbred lines, Ragot et al. (1995) recovered 99.3% RPG in four BCs [4]. Similarly, by screening 1,017 BC plants with 95 markers in an introgression effort for the rice submergence tolerance QTL *Sub1*, 95% RPG was recovered in just two backcrosses [5]. Due to lack of agronomic performance data and an accurate linkage drag assessment, it is difficult to determine the success of that experiment. Although these examples indicate that the technique has promise, but a systematic approach for optimizing various variables for MABS is lacking, especially in wheat.

There are four main MABS variables: (i) number of backcrosses, (ii) population size, (iii) number and spacing among markers, and (iv) number of marker data points (MDPs) (calculated as per marker per plant). Double crossover identified using markers flanking 1 cM of the target gene in a single BC cycle was estimated to require ∼94,000 plants compared to 2,000 plants required to accomplish the same using a step-wise marker-based selection process for two backcross generations [6]. An RPG recovery of ∼96% was estimated to require 2,280 MDPs on 40 plants during three BC cycles compared to 10,100 MDPs on 200 plants in two BC cycles [7]. Similar simulations are available for the number and spacing of markers, and for number of BC cycles. However, a weighted simulation including all variables has not been conducted to identify the ideal MABS approach.

Available simulations are based on several faulty assumptions. The physical size of chromosomal segments in linkage drag will depend upon the recombination rate around the target gene. The assumption of a uniform distribution of genes and recombination rate on chromosomes is grossly incorrect [8]. Distribution of recombination is highly uneven across chromosomes of higher eukaryotes [8]. In wheat, there are on average four crossovers per chromosome that are mainly restricted to the 48 gene-rich regions (GRRs) that account for more than 85% of the genes but span less than 29% of the genome [9]. The recombination rate even among various GRRs varies as much as 140-fold. Furthermore, the recombination rate within a particular GRR can vary more than 10-fold [8]. Incorporating genome structure information should make the simulation models more accurate.

The objective of this study was to develop and optimize an efficient, accurate and rapid method of introgressing genes into popular genetic backgrounds. First, computer simulations were performed to determine the optimal level of each of the four MABS variables by incorporating the available information on genome structure into the model and the distribution of genes and recombination rates on wheat chromosomes. The simulation results were used to develop a backcrossing scheme, the efficiency of which was tested by transferring a stripe rust resistance gene *Yr15* into a susceptible but otherwise very good soft white spring wheat cultivar ‘Zak’. A revised MABS approach was then proposed where each of the steps were empirically optimized.

## Results

### Comparison of Various MABS Strategies by Computer Simulations

**Figure 1:**
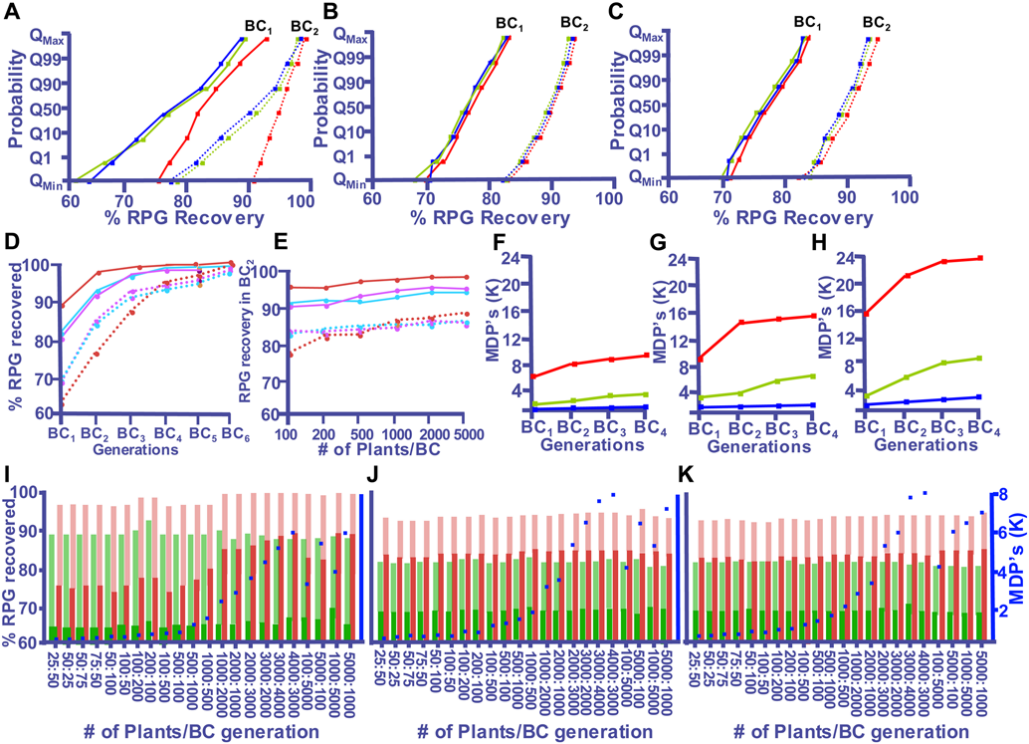
Computer simulations to optimize MABS approach in wheat. (A–C) Plabsim results for RPG recovery at different Q-values including the minimum and maximum value using 110 (A), 208 (B) and 320 (C) markers for two-stage (red line), three-stage (green line) and four-stage (blue line) selection. (D–E) Represents %RPG recovered with increasing BC generation at constant population size of 100 per BC (D), and with constant increase in population size (E), using 110 (red line), 208 (blue lines) and 320 (pink lines) markers and four-stage selection strategy. Solid lines corresponds to Q_max_ values, whereas dotted lines represents Q_min_ values. (F–H) Corresponds to the number of MDP required in each generation using two- (red line), three- (green line) and four-stage (blue line) selection strategy for 208 (F), 110 (G) and 320 (H) markers. (I–K) Represents the Q_max_ (shaded bars) and Q_min_ values (solid bars) of RPG recovery in BC_1_ (green bars) and BC_2_ (red bars) generation using variable population size per BC and four-stage selection, and for 110 (I), 208 (J) and 320 (K) markers. Blue dots correspond to the number of MDPs required.

Recurrent parent genome (RPG) recovery is the main output of the ‘Plabsim’- based simulations (methods) and it varies at different probability levels. Q_1_ represents a probability of 0.99 that a specific percentage of RPG will be attained, whereas Q_99_ represents a probability of 0.01. Q_min_ and Q_max_ are the maximum and minimum probabilities, respectively, flanking the probability range of Q_1_ to Q_99_ (Figure 1a–c).

Simulations were performed for two-, three- and four-stage MABS approaches. Selection in the two-stage approach was for the target gene followed by a genome-wide marker analysis to identify a plant carrying the maximum number of recurrent parent alleles. The three-stage approach was similar to the two-stage, except for an extra step to recover a double recombinant around the target gene preceding the genome-wide marker analysis. The four-stage approach had an extra step of marker analysis for the rest of the carrier chromosome following the recovery of a double recombinant [7]. Simulations were performed using 110, 320, and 500 markers, although no results were obtained with 500 markers due to computer memory limitations. To incorporate genome structure information into simulations, 208 DNA markers were selected (methods). Commonly assumed backcross expectations [1] were used as simulation controls.

Average RPG recovery after BC_1_ is expected to be 75% [1]. With an average of 74.9%, the RPG recovery for the control simulation with 208 markers ranged from 68.5% at Q_min_ to 82% at Q_max_. Similarly for BC_6_ the average simulated RPG recovery was 99.1% compared to the expected 99.2%. The mean RPG recovery for 110 and 320 markers after BC_6_ was 98.4% and 98.9%, respectively. These results for the controls validate the simulation models as the observed and the expected numbers were very similar.

To study the effect of marker number on RPG recovery, simulations of 110 markers at an average spacing of 23 cM were compared with that of 320 markers spaced at an average distance of 8 cM. These simulations were then compared with those performed using the 208 selected markers (see material and methods section), and the results at different probabilities (Q values) are provided in Figures 1a–c. In general, there was an inverse relationship between RPG recovery and probability value. The RPG recovery was the highest at the lowest probability (Q_max_ value). With 110 markers, differences in RPG recovery among the probability levels were highest with 33% in BC_1_ and 24% in BC_2_ (Figure 1a–c). RPG recovery rates were reduced by more than 50% when either 208 or 320 markers were used. This pattern of differences among probabilities was similar for all three-selection strategies used.

Among selection strategies, the difference in RPG recovery at various Q values was very small for the two-stage selection (range of 91 to 98%) compared to either three- or four-stage selection, where the range was 78 to 96%. Unexpectedly, RPG recovery was lower with three- or four-stage selection compared to the two-stage selection. In the BC_2_ generation, for example, the two-stage selection simulated a recovery of 91% of the RPG compared to 78% with the four-stage selection (Figure 1a). These differences were essentially non-existent when either 208 or 320 markers were used (Figure 1b–c). Among the three selection strategies, the difference in RPG recovery was more pronounced at lower Q values, with maximum difference observed with 110 markers compared to 208 or 320 markers. In the BC_1_ generation, the Q_min_ value at the two-stage selection with 110 markers was 77% compared to 60 and 62.5% for the three- and the four-stage, respectively, whereas the Q_max_ value was 91% for the two-stage compared to 89.5% for both the three- and the four-stage selection (Figure 1a).

The number of markers evaluated had a significant effect on the RPG recovery. At the Q_max_ value and using 100 plants per BC generation, RPG recovery in BC_2_ generation was simulated to be 98% when using 110 markers (Figure 1d). The same level of RPG was possible only in BC_4_ generation if 208 or 320 markers were used per backcross cycle (Figure 1d). Surprisingly, population size showed very little effect on RPG recovery. Only a slight increase from 92.5% to 94% was simulated by increasing the population size from 100 to 2,000 plants per backcrossing generation with 208 markers (Figure 1e). Increasing the population size to 5,000 did not increase RPG recovery.

As was seen for the constant size of population in each generation, the RPG recovery with variable population size over generations was higher with 110 markers compared to that with 208 or 320 markers (Figure 1i–k). With 110 markers, predicted RPG recovery (Q_max_ value) was also higher when larger population sizes were used in the BC_2_ generation (Figure 1i). For example, the RPG recovery with a 100BC_1_∶500BC_2_ population size was 99% in BC_2_ compared to 97.5% with 500BC_1_∶100BC_2_ population size. No marked difference in RPG recovery was observed between 208 and 320 markers (Figure 1j–k). With one exception, variable population size for different BC generations had a marginal effect on RPG recovery (Figure 1j). A population size of 5,000 in BC_1_ followed by 100 in BC_2_ simulated an RPG recovery of 94.9% (Figure 1j).

The most dramatic difference among various selection approaches was for the required number of marker data points (MDPs) (Figure 1f–h). For a given RPG recovery (∼93%) using 208 markers, the four-stage selection required 745 MDPs in BC_2_ generation compared to 13,060 for the two-stage selection. This difference in MDPs increased dramatically with the increase in population size. The difference in required MDPs between the two- and the four-stage selection was ∼16 fold for a population size of 100 compared to 48 fold for 5,000 plants. Because it required the least number of MDPs for a given RPG recovery, the four-stage selection was identified to be the most efficient MABS approach.

The three selection approaches (two-, three- and four-stage selection) were also compared for the number of MDPs required when using different marker numbers. Keeping the population size (100 plants/BC) and the RPG recovery constant, selection using 110 markers required the least number of MDPs compared to 208 or 320 (Figure 1f–h). For two-stage selection, the increase in MDPs over 110 markers was 2 fold for 208 markers and 3 fold for 320 markers, whereas with the four-stage selection this increase was 2 fold for 208 and 320 markers. As expected, the number of required MDPs increased with the increase in population size. This increase in MDPs was the largest for two-stage selection followed by the three- and the four-stage selection schemes. Using 110 markers in BC_2_ generation, a population increase from 100 to 5,000 plants increased the required number of MDPs for the two-stage selection from 7,981 to 372,256, whereas a similar increase with the four-stage selection was only from 410 to 7,832. By comparing various variable sized populations, the required number of MDPs was higher if a larger population was used in BC_1_ generation compared to the reverse (Figure 1i and k). However, with 208 markers and a population size of <500, a reverse trend was observed. The number of required MDPs for a population size of 75BC_1_∶50BC_2_ was 621, compared to 718 MDPs for a population size of 50BC_1_∶75BC_2_.

### Introgression of the Yr15 gene into the cultivar ‘Zak’

**Figure 2:**
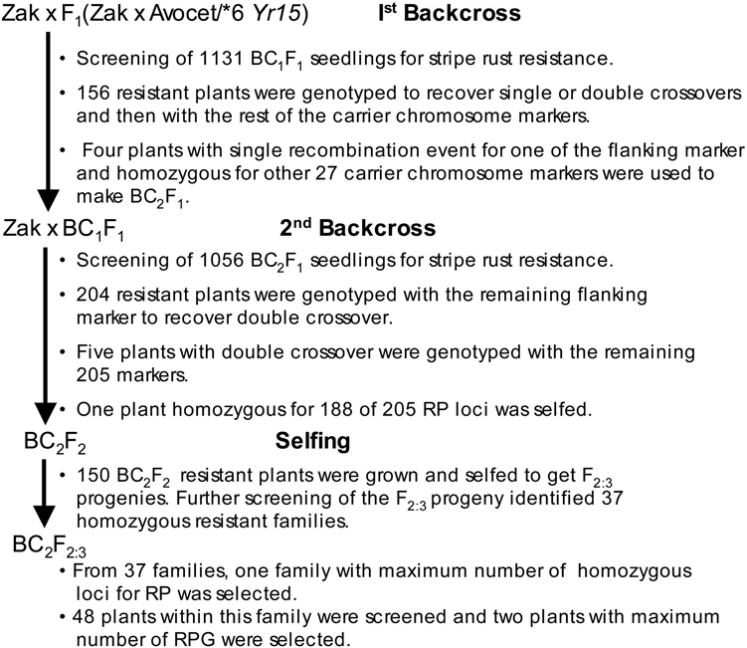
Schematic representation of introgression of stripe rust resistance gene *Yr15* into the cultivar ‘Zak’ using MABS.

Using the simulation results, as well as information on wheat genome structure [9], a four-step MABS approach was devised (Figure 2) and was used to transfer *Yr15* stripe rust resistance gene from ‘Avocet S*6/*Yr15*’ to a susceptible soft white spring wheat cultivar ‘Zak’. The efficiency of this approach was evaluated by comparing results to a control where no marker-assisted background selection was performed to transfer the same gene into ‘Zak’.

Segregation for the *Yr15* gene was distorted in all of the BC generations; however, the F_2_ population had the expected 3∶1 segregation ratio. Stripe rust screening of 1,131 BC_1_F_1_ plants identified only 156 (Chi-square [χ^2^] = 296, *P*-value<0.001) resistant plants instead of the expected 565 (1∶1 ratio). Similar screening of 1,056 BC_2_F_1_ plants identified 204 resistant plants (χ^2^ = 199, *P*-value<0.001). *Yr15* gene segregation also was distorted in a reciprocal BC_1_F_1_ population, as only 24 of the 90 plants were resistant (χ^2^ = 9.8, *P*-value<0.001). However, a screening of 76 F_2∶3_ families from the same cross displayed the expected 1∶2∶1 ratio (χ^2^ = 0.32, *P* = 0.85).

The two parents were subjected to a polymorphism survey with 639 simple sequence repeat (SSR) markers and the results are presented in Table 1. Approximately 49% (314/639) of the analyzed markers detected polymorphism among the recurrent and donor parent. For the *Yr15* gene carrier chromosome 1B, 29 of the 55 markers were polymorphic. Of these 29, 10 were on the carrier arm (short arm). The marker *Xgwm33* flanks the target gene *Yr15* on the distal side and *Xgwm11* on the proximal. Marker *Xgwm413* is the most tightly linked to the target gene but its orientation relative to the other two flanking markers was unknown.

**Table 1:** Polymorphism survey of ‘Zak’ and the *Yr15* gene donor line using SSR markers.

Wheat Chromosome	Polymorphic/Analyzed	Polymorphic/Analyzed	Polymorphic/Analyzed	Polymorphic/Analyzed
	A	B	D	Total
1	14/35	35/55	18/42	67/132
2	24/52	8/24	15/33	47/109
3	29/49	30/47	20/49	79/145
4	10/28	6/16	12/22	28/66
5	24/47	10/23	13/28	47/98
6	6/16	9/18	6/12	21/46
7	7/10	6/12	12/21	25/43
**Total**	**114/237**	**104/195**	**96/207**	**314/639**

Genotyping of the 156 resistant BC_1_F_1_ plants with *Xgwm33* identified 12 that were homozygous for the recurrent parent allele (RPA). Genotyping of these 12 plants with the remaining 28 1B-specific markers identified two that were homozygous for all but one RPA, the flanking marker *Xgwm11* for which all 12 plants were heterozygous. Two other plants also were very similar except that they were heterozygous for the linked marker *Xgwm413*. These four plants were used as male parents to generate 1,056 BC_2_F_1_ plants, of which only 204 showed immune response when screened with rust race PST-78. Genotyping of these resistant plants with the flanking marker *Xgwm11* identified five that were homozygous for the marker. Genotyping of these five plants with the remaining 205 selected markers (described earlier) identified a plant that was homozygous for RPAs of 188 markers and was heterozygous for the remaining 17 (Table 2). Stripe rust screening of 300 of its selfed progeny (BC_2_F_2_) identified 150 resistant plants, of which 37 were confirmed to be homozygous resistant upon screening of their F_2∶3_ progeny. These 37 families were genotyped with the 19 markers for which the four parent BC_1_ plants were heterozygous, and a plant homozygous for the maximum number of RPAs was selected. Further genotyping of 48 of its selfed progeny identified a plant (designated ‘WA8059’) that was homozygous for 243 of the 251 loci (97% RPG). ‘WA8059’ was selected for field evaluation.

**Table 2:** Recurrent parent genome (RPG) recovery by MABS.

Loci Analyzed*	BC_1_F_1_	BC_2_F_1_	BC_2_F_2∶3_
Number of loci analyzed	115	205	251
Homozygous loci for RP	84	188	243
Homozygous loci for DP	0	0	2
Heterozygous loci	31	17	6
Percentage of RPG	73	92	96.8
Percentage of DPG	0	0	0.8
Percent Heterozygous loci	27	8	3.2

* DPG: Donor parent genome; RP: Recurrent parent; DP: Donor parent.

### Comparison of backcross breeding with and without MABS

**Figure 3:**
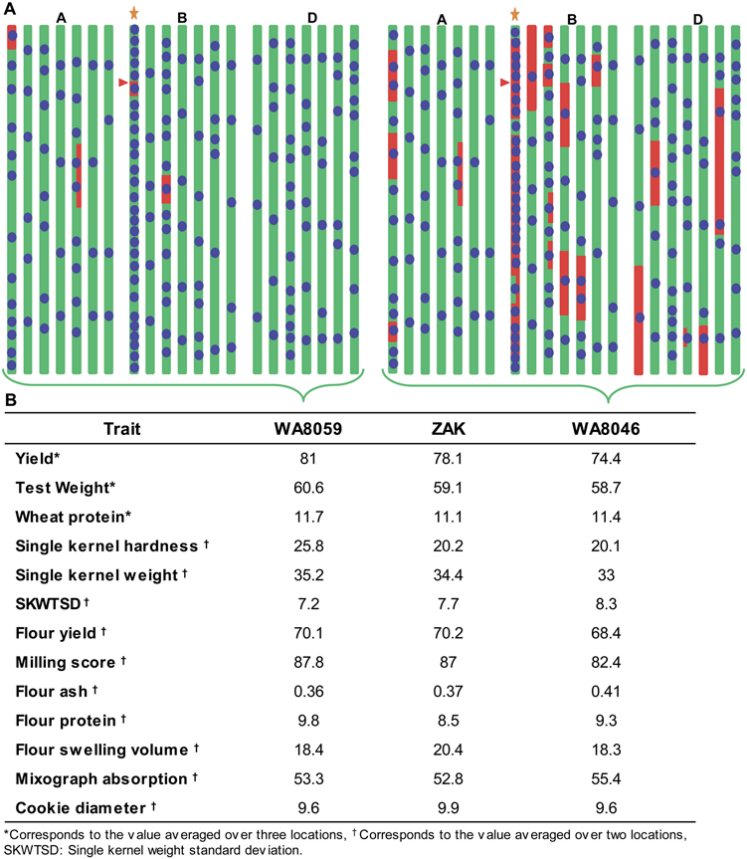
Comparison of cultivar ‘Zak’ derivatives carrying stripe rust resistance gene *Yr15* developed with (WA8059) and without (WA8046) MABS. (A) Graphical genotypes, showing parental derivation with green bars representing the homozygous RP alleles, red bars indicate the marker allele homozygous for DP, and heterozygous loci were marked with half green and half red bars. Orange star represents the carrier chromosome, with red arrow corresponding to the approximate position of the target gene (*Yr15*) on the carrier chromosome and blue dots denotes relative position of the SSR markers used for the comparison. (B) Comparison of various parameters recorded during field evaluation and quality analysis of these three lines done at different locations.

The MABS derived line ‘WA8059’ was compared with a BC_4_F_7_ line ‘WA8046’ that was developed using the same two parents, but by performing backcross breeding with phenotypic selection but without MABS. Genotypic comparison between ‘WA8059’ and ‘WA8046’ was performed with the same set of polymorphic SSR markers that were used for the MABS. The results with 236 SSR markers showed that ‘WA8059’ contained 97% of the RPG compared to 82% in ‘WA8046’ (Figure 3a). The largest difference was for the carrier chromosome (1B) where ‘WA8059’ contained 96.5% of the RPG compared to only 21% in ‘WA8046’. The RPG percentage for the non-carrier chromosomes was 90% for ‘WA8046’ compared to 97.1% for ‘WA8059’.

Grain yield and quality comparisons were performed by growing ‘WA8059’, ‘WA8046’, and the recurrent parent ‘Zak’, under Washington State University (WSU) uniform cereal variety testing trail at 17 locations during the 2007 crop year (http://variety.wsu.edu/2007/index.htm#annualreport) (Figure 3b). The data averaged over 17 locations, suggested that the line ‘WA8059’ derived using MABS was very similar to the recurrent parent ‘Zak’, with average yield (bu/ac), test weight (lbs/bu) and grain protein content (%) of 51.0, 60.0 and 12.7 compared to 51.7, 58.8 and 12.1 for ‘Zak’ and 50.3, 58.8 and 12.0 for ‘WA8046’, respectively (http://variety.wsu.edu/2007/index.htm#annualreport). The ANOVA for test weight of ‘WA8059’ was significant at *P* = 0.057. The MABS derived line ‘WA8059’ also exhibited more uniform kernel weight as evidenced by a significantly lower kernel weight standard deviation (Figure 3b). Averaged over the locations where ‘Zak’ was typically grown (>20″ rainfall) ‘WA8059’ showed a 4% increase in grain yield over ‘Zak’ and 9% over ‘WA8046’. Under high rainfall conditions like Pullman, Washington, the grain yield advantage over ‘Zak’ was 19.2%. Stripe rust infection type scored at the scale of 0–9, with 0–3 considered as resistant, 4–6 intermediate, and 7–9 susceptible was adequate for all locations for reliable data (http://variety.wsu.edu/2007/index.htm#annualreport). Infection type of ‘Zak’ at different locations ranged from 5 to 8, whereas both ‘WA8059’ and ‘WA8046’ showed a resistant reaction of 2 at all locations observed. In general the grain quality characteristics of ‘WA8059’ were similar to that of ‘Zak’ but subtle differences were observed. In particular, line ‘WA8059’ showed slightly increased flour protein (FPROT) and milling score (MSCOR) over both ‘Zak’ and ‘WA8046’ (Figure 3b), whereas grain of ‘WA8059’ was slightly harder than both ‘Zak’ and ‘WA8046’ as the single kernel hardness (SKHRD) value was 28% higher but all values were well within the range typical for Pacific Northwest (PNW) soft white wheat. All other quality characteristics were similar among the three lines.

## Discussion

Here we report a quick, precise, and efficient method of marker-assisted background selection (MABS) to transfer targeted genes among wheat cultivars or germplasms. With the number of plants per backcross (BC) cycle being the only variable, it is possible to recover a high level of recurrent parent genome (RPG) in just two backcrosses. We found that reducing linkage drag on the carrier chromosome was the most difficult part of the procedure. As seen in the control line (WA8046), without MABS double crossovers for the markers flanking the gene of interest are less likely to be selected even after many BC cycles probably because of a very low frequency of occurrence. However, we were able to recover 97% of the RPG in just two BC cycles including 96.5% for the carrier chromosome using the four-stage MABS approach optimized in this study. Since the MABS-derived line had grain yields that were equal to or better than the recurrent parent (RP), the MABS approach may have eliminated the yield penalty that is typically associated with the standard backcross breeding because of a linkage drag.

Most likely demarcated by the Q_min_ and Q_max_ probabilities of Plabsim simulations, various plants in a BC population are expected to contain a wide range of RPG percentage [1]. Random selection of only a few BC plants is likely to identify plants with an average percentage of RPG and not the rare desirable plants with much higher RPG percentages. Even if selected by chance, without MABS the desirable plant will not be retained on priority and will most likely be mixed with other selected plants containing an average RPG percentage. Perhaps this is the reason why our MABS approach recovered such a high percentage of RPG in just two BC cycles compared to the control.

As predicted by simulations and demonstrated by our results, recovering the carrier chromosome is the biggest challenge during BC breeding. If 1 cM apart, only one out of 100 plants is expected to show recombination between a marker and the target gene (Table 3). Recombination will occur in one out of ten plants if the marker is 10 cM from the target gene. Assuming no chiasma interference, a double recombinant for markers present at 1 cM flanking the target gene in a single BC generation will require systematic screening of 10,000 plants (Table 3). The same number increased to 24,000 when simulations using Popmin software were performed [10]. Therefore, sequential identification of a double crossover in two BC cycles compared to selection in a single BC cycle would require a much smaller number of plants. For flanking markers at 1 cM from the target gene, one out of 50 plants is expected to be recombinant for at least one of the two markers. For the second marker in the following BC cycle, however, the probability will be 1/100. Selecting for the remaining carrier chromosome markers should be relatively easy as the recombination rate increases proportionally to the genetic distance from the target gene. However, multiple double recombinant plants will be needed to facilitate this selection although it is difficult to estimate an exact number.

**Table 3:** Number of plants required to recover single and/or double recombinant (Rec.) with desired physical size.

Flanking Markers (cM)	Number of Plants/BC_1_				Size of Linkage Drag (Mb)		
	One Gene		Two Gene		Recombination in kb/cM		
	Single Rec.	Double Rec.	Single Rec.	Double Rec.	150–5,000	5,000–10,000	10,000–22,000
1	50	10,000	2,500	100,000,000	0.15–5	5–10	10–22
2	25	2,500	625	6,250,000	0.3–10	10–20	20–44
4	13	625	156	3,90,625	0.6–20	20–40	40–88
6	8	278	64	77,284	0.9–30	30–60	60–132
8	6	156	36	24,336	1.2–40	40–80	80–176
10	5	100	25	10,000	1.5–50	50–100	100–2200

Difficulty in removing linkage drag on the carrier chromosome was exemplified in our results. Without the MABS, 79% of the donor chromosome 1B (carrier chromosome) was retained in the BC_4_F_7_ derived line ‘WA8046’ (Figure 3a). The same number for the BC_2_F_3_ line (WA8059) developed by MABS of 360 BC plants with 29 carrier chromosome-specific markers was only 3.5%. Phenotypic selection of plants for four BC cycles followed by seven selfing cycles did not result in the predicted (96.9%) amount of RPG as only 82% of the marker alleles were from the recurrent parent. Even for the non-carrier chromosomes the RPG recovery was 90%. With MABS however, 97% of the RPG was recovered with six fewer generations (BC_2_F_3_ vs. BC_4_F_7_) with essentially no difference between carrier and non-carrier chromosomes. The marker analysis results were validated by field evaluation results, and the yield and quality trials for the MABS line were either equal to or better than the recurrent parent ‘Zak’.

The average expected RPG recovery is 75% in BC_1_ and 87.5% in BC_2_ generation [1]. Although these numbers are accurate for the total RPG proportion, average number of RP homozygous chromosomes per plant is significantly lower than expected. Reflecting gametic proportions in the F_1_, average number of RP homozygous chromosomes per plant is expected to be 25% (∼5 of the 21 chromosomes) with a full range from 0 to 100% due to independent assortment of chromosomes. The probability of a BC_1_ plant to be RP homozygous for all 21 chromosomes is 2.2×10^−13^ (0.25^21^). In addition to the chromosomes that were fixed in BC_1_, the probability of the remaining chromosomes being RP homozygous may approach 0.5 in BC_2_. About 25% of the chromosomes in a BC_1_ gamete will be RP type and additional 25% of RP type chromosomes will be generated by recombination. With that probability, approximately seven of the remaining 15 chromosomes are expected to the RP homozygous in each of the BC_2_ plants. Therefore, a plant RP homozygous for all chromosomes should be present among 256 BC_2_ selected plants (0.5^8^) carrying the target gene. Each BC_1_ plant is expected to carry 25% RP homozygous chromosomes and the probability of RP homozygous chromosomes in BC_2_ generation is expected to be up to double than that in BC_1_ generation, therefore, background selection for the non-carrier chromosomes should be delayed until BC_2_ generation. Since the carrier chromosome is more difficult to deal with, the main objective in the BC_1_ generation should be to recover as much of the carrier chromosome as possible. Our results on the MABS-based introgression of the *Yr15* gene agree with the above interpretations and estimations. Screening of approximately 1,000 plants per BC cycle resulted in a recovery of 96.5% of the carrier chromosome. For the non-carrier chromosomes, however, MABS of only four selected plants in BC_1_ and five in BC_2_ resulted in a recovery of 97.1% of the non-carrier genome.

Comparison of the results from empirical testing of the MABS strategy did not consistently align with prediction based on computer simulations. Predicted by the simulations to be the most efficient, the four-step selection strategy was very effective for transferring the *Yr15* gene and recovering 97% of the RPG in just two BC cycles (Table 2). In contrast, the simulations failed to establish the expected relationship between RPG recovery and population size. Shown by the Q_min_ and Q_max_ ranges using the four-stage selection and 100 plants per BC, RP content was simulated to range from 69.6% to 82.5% for the BC_1_ population and 82.2% to 92.5% for the BC_2_. Probability of finding a rare recombinant or a plant RP homozygous for the desired number of chromosomes should be directly proportional to the number of plants used for the MABS screening, such that any targeted goal of RPG recovery should be possible even in a single BC generation provided that a proportionally larger population is used. However, simulations results demonstrated very little gain in RPG recovery in response to an increase in population size. The RPG recovery (Q_min_ value) using 5,000 plants per BC was simulated to be 84.1% compared to 82.2% with only 100 plants. Essentially no gain in the RPG recovery was predicted by increasing population size from 2,000 to 5,000 plant/BC cycle (Figure 1e).

Empirically the four-step MABS approach resulted in a higher RPG recovery than what was predicted by simulations. Using 200 plants per BC cycle, the maximum (Q_max_) RPG recovery was simulated to be 93% and that only increased to 93.7% by increasing the population size to 1,000. We were able to recover 97% RPG using about 1,000 plants per BC generation, and the effective population size was about 25% of that due to the distorted segregation of *Yr15* gene. Only about 17% of the BC plants were resistant to stripe rust instead of the expected 50%.

Since the number of MDPs is a very important factor for MABS, it is imperative to determine a minimum number of markers required to recover a desired RP percentage. The Plabsim simulations predicted a higher RPG recovery with 110 markers compared to 208 and/or 320 markers, whereas no marked difference was predicted between 208 and 320 markers (Figure 1d and e). This difference between 110 and 208 markers is most likely due to the predicted double crossovers that are more likely to be detected as marker number increases. The lack of difference between 208 and 320 markers suggests that either all the recombination events could be detected with 208 markers such that an increase in marker number to 320 did not demonstrate any gain. Alternatively, incorporating wheat genome structure information into the marker selection scheme increases detection power of 208 markers making them equivalent to 320 markers. It is difficult to differentiate between these two possibilities.

During the introgression of *Yr15* gene, the use 236 markers for the MABS approach produced an RPG recovery of 97%. If the same analysis was performed using 100 markers randomly selected from 236, the RPG recovery was 97.2% (data not shown). Only two additional donor segments, each detected by a single marker, were detected by adding 136 more markers. Results suggest that perhaps 100 selected markers (about two per arm) (see methods section for marker selection scheme) are sufficient for the proposed MABS approach although additional markers will further increase accuracy of the RPG recovery estimates for the selected plants. This will be particularly true for the MABS approach described below where the main focus is to select non-recombinant, non-carrier chromosomes rather than depending upon the recombinant chromosomes that may have small introgression segments.

Sequential marker analysis of the control line ‘WA8046’ first with the 100 markers and then with the additional 136 markers, identified 24 donor parent (DP) segments that were not identified by the 100 markers (data not shown). Compared to ‘WA8059’ (BC_2_F_3_) where only four cycles of recombination were allowed, ‘WA8046’ (BC_4_F_7_) underwent 10 cycles of recombination. Additional recombination cycles are expected to result in smaller-sized donor segments that are less likely to be detected with fewer markers. Fewer recombination cycles as was the case for ‘WA8059’ probably resulted in larger donor segments that were easily identified during MABS and thus were selected out. Lack of MABS in ‘WA8046’, especially during the earlier generations, allowed the donor segments to be selected that were then broken into smaller segments during the consecutive recombination cycles.

It is imperative to know the structure and recombination rate of the region containing the target gene in order to accurately design the MABS approach and to interpret the results. Recombination in wheat mainly occurs in the gene-rich regions that account for less than 28% of the genome [9]. Even among various gene-rich regions the recombination rate varies up to 140 fold, with 1 cM translating to 151 kb per cM for the gene rich region ‘1S0.8’ compared to 21,687 kb per cM for ‘7S0.8’ (Table 4). With a target gene present in a recombination high region like ‘1S0.8’, 100 plants per BC may reduce the linkage drag to 151 kb, whereas 5,000 plants per BC may reduce it further to about 3 kb. However, with the target gene in a recombination poor region like ‘7S0.8’, the size of the linked donor segment can be up to 21 Mb with 100 plants per BC and 216 kb with 10,000.

**Table 4:** Physical Locations of Genes/Markers, recombination (Rec.) in GRRs in Wheat Genome.

GRRs	Genes (%)	Rec. (%)	Size (Mb)	Rec. in kb/cM
‘1S0.8’	71	86	7	151
‘1S0.6’	20	8	15	3488
‘1S0.4’	9	3	3	2143
‘1L0.1’	18	3	17	6071
‘1L0.4’	16	14	21	1280
‘1L0.7’	22	21	35	1440
‘1L0.9’	31	43	14	280
‘1L1.0’	11	8	7	753
‘2S0.9’	46	37	39	1300
‘2S0.8’	31	39	7	215
‘2S0.5’	23	17	21	1500
‘2L0.3’	9	4	44	8800
‘2L0.5’	23	17	9	450
‘2L0.8’	10	4	36	7200
‘2L1.0’	58	68	22	275
‘3S0.9’	39	36	46	1107
‘3S0.8’	31	39	25	580
‘3S0.5’	31	20	60	4332
‘3L0.3’	20	15	19	1382
‘3L0.5’	22	9	22	1850
‘3L0.8’	14	10	14	609
‘3L0.9’	44	66	71	490
‘4S0.9’	30	24	53	4400
‘4S0.7’	70	61	41	1350
‘4L0.5’	38	15	70	6350
‘4L0.7’	22	12	37	4000
‘4L0.9’	40	68	60	1220
‘5S0.9’	29	60	43	2389
‘5S0.7’	39	28	21	3000
‘5S0.6’	24	5	19	6333
‘5S0.4’	14	3	13	4333
‘5L0.3’	10	8	45	4091
‘5L0.5’	36	20	61	2033
‘5L0.8’	36	40	20	308
‘5L0.9’	11	29	66	1650
‘6S1.0’	82	89	10	164
‘6S0.5’	18	9	41	6949
‘6L0.4’	30	21	15	847
‘6L0.7’	10	6	60	11765
‘6L0.9’	60	72	45	825
‘7S0.9’	30	32	40	1481
‘7S0.8’	23	22	36	1978
‘7S0.4’	31	43	29	853
‘7S0.2’	10	2	36	21687
‘7L0.1’	3	1	33	15278
‘7L0.3’	19	5	45	13889
‘7L0.8’	43	39	41	863
‘7L1.0’	31	53	21	442

This table is adapted from Erayman et al. 2004 [9].

### Proposed MABS strategy

**Figure 4:**
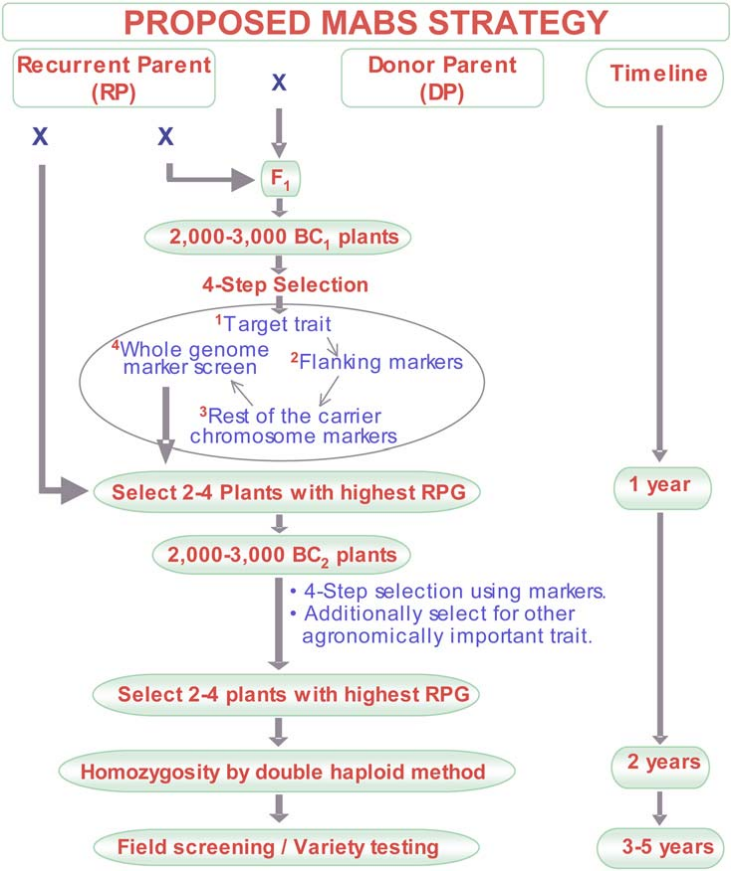
Proposed MABS strategy in wheat for introgressing a target gene while recovering 97% or more RPG in just two BCs.

**Figure 5:**
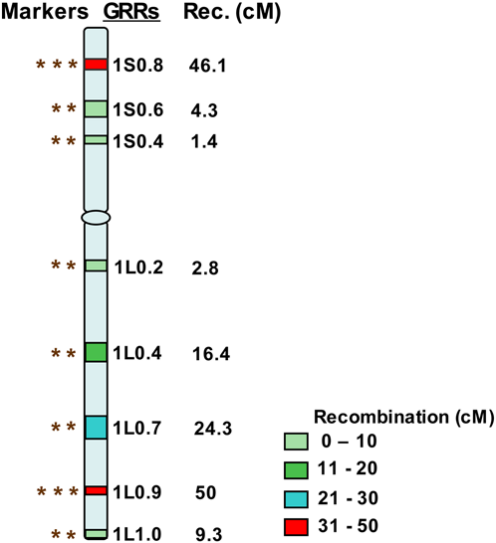
Strategy for selection of markers for marker-assisted backcross breeding program. Example is given for consensus chromosome of group 1. Names of the GRRs are provided on the right side of a consensus chromosome. In the nomenclature of GRRs (e.g. ‘1S0.8’), the first digit represents wheat homoeologous group followed by the arm location either as short arm (S) or long arm (L). The last two numeral numbers represent GRR location as fraction length (FL) of the chromosome (e.g. 0.8 for ‘1S0.8’). Along with GRRs the recombination in cM observed for particular GRRs is also given. On the left side of the consensus chromosome, * denotes the number of markers selected for a particular GRR region. Number of markers selected for each GRR was variable. For example the regions showing high recombination, like GRR 1L0.9, three markers were selected, two flanking and one from the middle of the GRR, whereas in the case of a GRR like 1S0.4, only two markers flanking the GRR was selected.

By taking into account the theoretical calculations, computer simulations, the *Yr15* gene transfer results and experiences, and the structural and functional organization of the wheat genome, we are proposing the following four-stage MABS strategy for gene transfers in wheat (Figure 4). Markers for the procedure should be selected using the information on the distribution of genes and recombination, as shown in the methods section (Figure 5). As mentioned earlier, markers on the carrier chromosome should have an average spacing of 5 cM (about 25 to 40 polymorphic markers). For the non-carrier chromosomes, two markers per arm are adequate, although additional markers may further increase accuracy of the RPG recovery estimates.

The required number of plants per BC cycle depends upon the distance of the flanking markers from the target gene, recombination rate in the chromosomal region, and desired level of homozygosity in the selected plants (Table 3 and 4). Instead of seeking it in a single generation, a double recombinant should be sequentially attained over two BC cycles. Recombination between the target gene and one of the flanking markers should be identified in BC_1_ and for the second flanking marker in BC_2_. The total number of plants needed to select a plant with recombination between the target gene and one of the flanking markers, is given in Table 3. With flanking markers at 4 cM from a target gene, the 10 recombinant plants can be selected out of 130 plants, whereas for the distance of 1 cM, 500 plants will be required. For a gene that is present in a region with a higher recombination rate, using flanking markers at 4–6 cM may be adequate. However, for a target gene present in a region with a lower recombination rate, all efforts should be made to use flanking markers as close to the gene as possible.

The selected BC_1_ plants (∼10) should then be screened with the remaining carrier chromosome markers. Plants carrying the least amount of DP alleles present at the longest distance from the target gene should be selected. Depending upon the number of plants needed to make the required sized BC_2_ population, the selected plants should then be screened with non-carrier chromosome markers to select plants carrying the highest number of RP homozygous chromosomes.

Assuming six to eight RP homozygous chromosomes were fixed in the selected BC_1_ plants, about 100 plants with recombination between the target gene and the second flanking marker are needed to select a plant carrying most, if not all, RP homozygous chromosomes. These 100 selected plants should then be screened for the markers that showed DP alleles during marker screening in BC_1_. The selected plants should either be instantly made homozygous by double haploid (DH) method or by going through one or more self-pollination cycles. The DH approach is preferred if speed is desired, but the selected plants should not have DP segments on too many chromosomes otherwise a larger DH population will be required to identify a plant containing the desired amount of RPG. The DH approach is also preferred if more than one gene is being introgressed, since only one out of 16 plants will be homozygous for both target genes in BC_2_F_2_ compared to one out of four in the DH progeny.

For transferring two target genes, the required population size to recover the ideal carrier chromosomes increases dramatically (Table 3), although the non-carrier chromosome estimates are the same as that for a single gene transfer. If a transfer involves more than two unlinked genes, each set of two genes should be transferred independently. At the end of the BC_2_ cycle, the selected plant from each lineage should then be crossed to each other and the F_1_ should be subjected to DH production in order to select plants carrying all desired genes.

## Materials and Methods

### Plant materials

The recurrent parent for all introgression experiments was a soft white spring wheat cultivar ‘Zak’ [11] that is highly susceptible to the prevailing race of stripe rust (*Puccinia striiformis* Westend f. sp. *tritici*) in the Pacific Northwest (PNW) region of the United States. The donor was a near-isogenic line of the spring wheat cultivar Avocet (PI 464644) that carries *Yr15* gene introgressed from *T. dicoccoides* Koern [12]. The recurrent parent was always used as the female parent for all crosses.

### Screening plants for stripe rust resistance

Screening for stripe rust resistance was carried out in a growth chamber using freshly amplified urediniospores of race PST-78 of *P. striiformis* f. sp. *tritici*, a highly virulent race prevalent in the PNW [13]. Plants at the two-leaf stage were inoculated with spores mixed with talcum powder, as described by Chen and Line [14]. A fine mist of water was sprayed on the plants and high relative humidity was ensured by placing plants in a water-containing trays covered with a plexiglass box to accomplish a water seal. After 16 hrs of incubation at 10°C, plants were moved to a second growth chamber with a temperature regime of 8°C and 16°C cycled every 6 hrs starting with 8°C at midnight. Disease scoring was performed after 21 days using the scale and method described by Chen and Line [14].

### High-throughput Marker analysis

A rapid, economical and efficient method of DNA extraction was optimized to extract sufficient amount of DNA to perform >200 polymerase chain reactions (PCR). For DNA extraction, two to four one-inch long leaf pieces were cut and placed into a 96-2 ml deep well plate (Axygen Scientific, Inc. USA) and lyophilized until leaves were completely dry (three to four days). Dried leaves were ground by adding a metallic bead (3 mm, V & P Scientific, USA) to each of the wells, followed by shaking in ‘Qiagen Mixer Mill’ (Model MM 301) (Qiagen, USA) at the maximum speed of 30 Hz for six min. Using a multi-channel pipettor, 750 µl of a solution containing 100 mM Tris pH 7.5, 1.4 M NaCl, 20 mM EDTA pH 8.0, 2% CTAB, and 0.2% Mercaptoethanol was added and the plates were incubated at 60°C for 60–90 min either by mixing (for higher yields) in a levitation machine (V&P Scientific, Inc. USA) or in a water bath. After chloroform∶octanol (24∶1) extraction, DNA was precipitated by adding 2/3 volume of isopropanol, and the pellet was washed in 70% ethanol; air-dried, and suspended in 400 µl of TE (500 mM Tris, 50 mM EDTA).

### Marker selection

Markers for background selection were selected using the strategy outlined in Figure 5, and by using gene and recombination distribution information provided by Erayman et al. (2004). Markers flanking each of the 48 GRRs were selected. For the GRRs with genetic distance larger than 45 cM, an additional marker from the middle of the region also was selected. For example, three markers were selected each for GRR 1S0.8 (46.1 cM) and 1L0.9 (50.0 cM), whereas only two flanking markers were selected for regions 1S0.6 (4.3 cM) and 1L0.4 (16.4 cM).

### Marker analysis

Markers for the project were selected out of the available list of wheat SSRs (http://wheat.pw.usda.gov/GG2/quickquery.shtml). The forward primers were synthesized with the M13 tail (CACGACGTTGTAAAACGAC) at the 5′ end in order to detect the PCR products on the IR2 4200 DNA Analyzer (Li-COR Biosciences, USA). The PCR reactions contained 25–100 ng of genomic DNA, 1× PCR buffer, 2.5 mM dNTP, 50 mM MgCl_2_, 1 pmol of IRDye M-13 forward tail primer (Li-COR), 1 pmol each of Forward and Reverse primers and 0.1 U *Taq* DNA polymerase in a 10 µL volume. PCR amplification was performed in 96-well plates on Px2 Thermal Cycler (Thermo Scientific, USA) with an initial cycle of 94°C for 3 min, 60°C for 1 min and 72°C for 1 min followed by 30 cycles of 94°C for 30 s; 60°C for 30 s; and 72°C for 30 s. The PCR product were stored in the dark at −20°C for up to 20 days. Before loading, the product was diluted with nine volumes of the loading dye (Formamide, 10 ml+Bromophenol Blue, 5 mg+0.5 M EDTA, 200 µl) and denatured at 94°C for 2 min and chilled on ice. Electrophoresis was carried out using IR2 4200 DNA Analyzer (Li-COR) on 6% polyacrylamide gel.

### Computer Simulations

Computer simulations were performed using Plabsim software [15], which is written in C++ and requires an input file in ASCII format. The input file containing the information regarding the genetic map, population size and design of the breeding program (ranging from two to six backcrosses) was written and run on Plabsim software installed on a computer with the *Unix* operating system, and 8GB RAM and Intel 2.33 GHz Xeon CPU. Different input files with varying population size and backcrossing generations were run on genetic maps with different marker numbers (110, 208, 320 and 500 markers). These maps were developed using a wheat SSR consensus genetic linkage map consisting of 1,235 markers spanning 2,569 cM [16]. The markers were uniformly placed at a distance of 23 cM for the map with 110 markers, 8 cM for 320 markers and 5 cM for 500 markers. In the case of the genetic map with 208 markers, 208 of the 251 polymorphic markers used for MABS were placed using the strategy shown in Figure 5. For all simulations, the target gene was assumed to be located ∼30 cM (to demarcate the carrier chromosome) from the telomeric end of chromosome 1BS with flanking markers at 2 cM interval on each side of the target gene. Each simulation was repeated 2,500 times.
